# Trees, shrubs and herbs of the coastal Myrtaceae swamp forest (Región de La Araucanía, Chile): a dataset

**DOI:** 10.3897/BDJ.9.e63634

**Published:** 2021-03-01

**Authors:** Jimmy Pincheira-Ulbrich, Elías Andrade Mansilla, Fernando Peña-Cortés, Cristián Vergara Fernández

**Affiliations:** 1 Laboratorio de Planificación Territorial, Departamento de Ciencias Ambientales, Facultad de Recursos Naturales, Universidad Católica de Temuco, Rudecindo Ortega 02950, Temuco, Chile Laboratorio de Planificación Territorial, Departamento de Ciencias Ambientales, Facultad de Recursos Naturales, Universidad Católica de Temuco, Rudecindo Ortega 02950 Temuco Chile; 2 Facultad de Educación, Universidad Católica de Temuco, Temuco, Chile Facultad de Educación, Universidad Católica de Temuco Temuco Chile

**Keywords:** biodiversity, cultural landscape, metacommunity, species richness, species inventory, wetlands

## Abstract

**Background:**

Species lists are fundamental for knowledge of species diversity in regions subject to intense anthropogenic pressure, especially in poorly-studied ecosystems. The dataset comes from an inventory conducted in 30 fragments of Myrtaceae swamp forest, located in an agroforestry matrix landscape of the coastal La Araucanía Region in Chile. The data collection was carried out using line transect sampling, which was traced through the core of each fragment orientated towards its longest axis. The dataset provides a record of 55 species (24 trees, 1 vine [as a host], 16 herbs and 15 shrubs) including accidental epiphytes (n = 7), hemiparasites (n = 4), host (n = 10) and additionally woody debris (n = 36). The most frequent trees in the landscape were *Myrceugenia exsucca* (n = 36 records) and *Blepharocalyx cruckshanksii* (n = 33 records), species that were also the most common hosts. *Drimys winteri* was a companion species, other trees and shrubs generally being rarely observed, as was the case of the introduced species (*Prunus avium*, *Rubus constrictus* and *Ulex europaeus*). Branches were the most common microhabitat for hemiparasites. Within this group, *Lepidoceras chilense* was the most frequent species. For accidental epiphytes, Drimys winteri, which commonly grows on the ground (soil), were the most common species found in the main trunk crotch. Some unusual observations were the climber *Cissus striata* as host of *Tristerix corymbosus* (hemiparasite) and *Tristerix corymbosus* as host of *Lepidoceras chilense* (hemiparasite).

**New information:**

This study represents a landscape-scale sample of the swamp forest, which is distributed in a dispersed pattern over a large stretch of Chile. The data were collected from 30 forest patches (from 0.05 to 936 ha), located on the coast of the Araucanía. The database includes the presence of 55 species of vascular plants in 356 records. The main novelty of this contribution is the systematic classification of species under six traits, never before reported in the same database: (i) condition (coarse woody debris, fallen log, live, snag), (ii) habit (herb, shrub, tree), (iii) growth microhabitat (e.g. tree trunk, branch, main trunk crotch), (iv) growth form (accidental epiphyte, hemiparasite, terricolous, vegetative), (v) host species (as appropriate) and (vi) relative location of the species in the sampled patch and surrounding areas (core, border, matrix). Species not previously observed in these forests were: *Gavilea* spp., *Hieracium* spp., *Lophosoria quadripinnata*, *Berberis actinacantha*, *Gaultheria phillyreifolia*, *Ovidia pillo-pillo*, *Amomyrtus meli* and *Caldcluvia paniculata*. In addition, two introduced species are novelties for the catalogue of vascular plants of Chile (*Cupressus macrocarpa* and *Prunus avium*). Several of these ecosystem traits are indeed new reports for these types of forests (e.g. accidental epiphytes, fallen logs, species-host relationship); at the same time, more frequent data (i.e. species composition, habit) are found in different contributions, making the comprehensive process of analysis difficult. Accordingly, the database is made available in this manuscript.

## Introduction

Species lists are fundamental for knowledge of species diversity in regions subject to strong anthropogenic pressure (Funk 2006, Hortal et al. 2007, Hermoso et al. 2013, Pincheira-Ulbrich et al. 2016, Cornwell et al. 2019).

South American swamp forests dominated by species of the family Myrtaceae Juss. are distributed in Chile in a dispersed pattern from 30ºS (Coquimbo) to 41º28'S (Puerto Montt), in a transition from semi-arid to temperate rain climate, representing one of the widest geographic and climatic distribution ecosystems in Chile (Maldonado and Villagrán 2001, San Martín 2005, Armesto et al. 2007, Ramírez et al. 2014).

To the north, their formations are located along the coast, while towards the centre and south of Chile, the patches are found both on the coast and in central plains and less frequently in the Andean foothills. Their location and extension are determined by the presence of surface phreatic layers, so the type of soil does not seem to be a determining factor in their growth. In this sense, these forests are described as azonal hydrophilic formations, meaning that their presence is not determined by the regional climate, but rather by an excess of edaphic humidity (see Amigo and Ramírez 1998, Maldonado and Villagrán 2001, Peña-Cortés et al. 2011, Ramírez et al. 2014).

Swamp forests are a particular type of wetland, classified by the Ramsar Convention as “forested freshwater wetlands”. At the international level, wetland areas have been recognised for their high biological and environmental value and as providers of ecosystem services (Zedler and Kercher 2005,Barbier 2013, Marton et al. 2015). In Chile, there is a Wetland Protection Policy expressed in the National Wetland Strategy and the National Biodiversity Strategy. However, swamp forests are one of the most altered ecosystems in this country, as they have frequently been threatened by human use pressure for agricultural fields, grazing and firewood extraction (San Martín et al. 1988, Squeo F et al. 2001, Ramírez et al. 2014).

These ecosystems are home to a rich diversity of vascular plants that varies from eight species in a highly-degraded site in central Chile (Ramírez et al. 2014) to 61 species in better-conserved sites in the central-southern zone of this country (Hauenstein et al. 2014). At a regional scale, the richness varies between 158 and 182 species, amongst landscapes in the northern area (San Martín et al. 1988) and south of their distribution (Larrain 2011), respectively. Along the coast of Araucania, these forests are mainly composed of *Myrceugenia exsucca* O.Berg and *Blepharocalyx cruckshanksii* (Hook. & Arn.) Nied. They are represented by an area of 7,675 ha, which is approximately 4.6% of this territory and where 427 forest fragments are distributed within a predominantly agricultural and forestry matrix. Forest is found exclusively in flat areas (alluvial plains), associated with both watercourses and different levels of soil waterlogging (Peña-Cortés et al. 2011).

**Figure 1.**
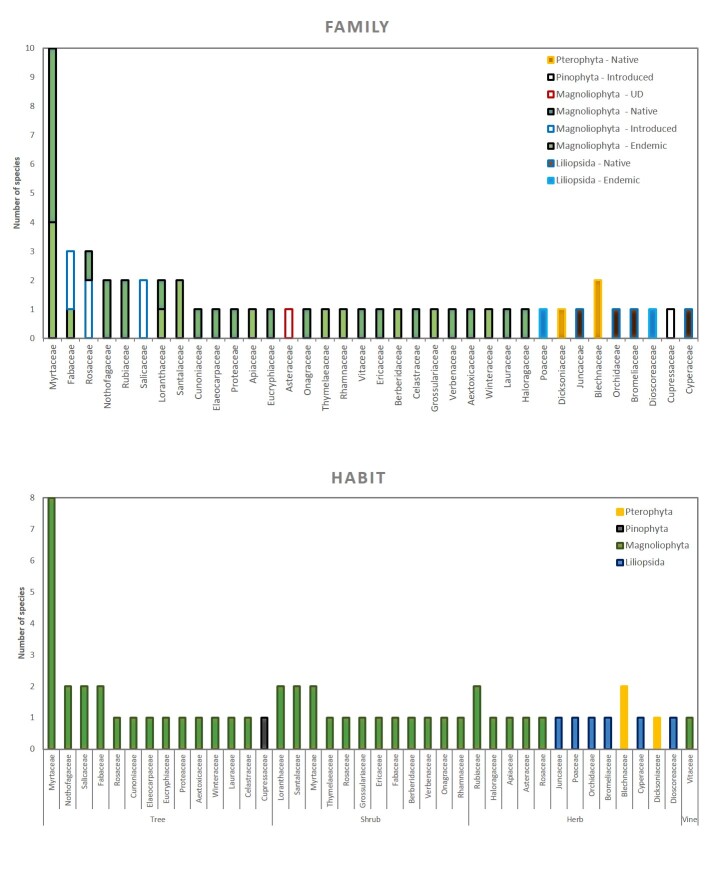
Species richness classified by number of families (top) and habit (bottom). In both figures, the species are arranged according to Phyllum, while in the upper figure, the geographical origin is also included.

**Figure 2.**
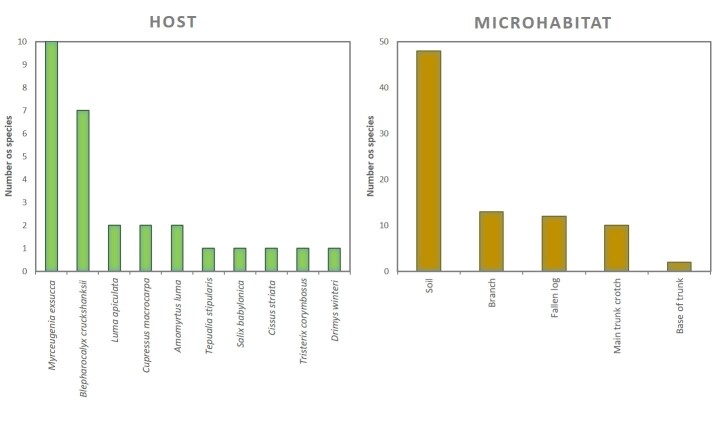
Species richness sorted by host (left) and microhabitat (right).

**Figure 3.**
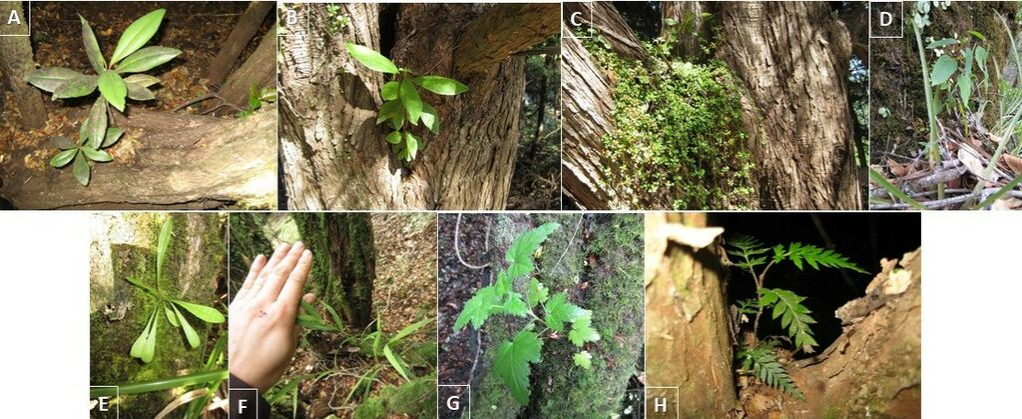
Accidental epiphytes: **A.** *Drimys winteri* growing on the base of a *Myrceugenia exsucca* trunk; **B.** *Drimys winteri* growing on *Cupressus macrocarpa* main trunk crotch; **C.** *Nertera granadensis* growing on *Cupressus macrocarpa* main trunk crotch; **D.** *Aristotelia chilensis* growing on *Blepharocalyx cruckshanksii* main trunk crotch; **E.** *Hieracium* spp. growing on the base of a *Myrceugenia exsucca* trunk; **F.** *Chusquea quila* growing on the base of a *Myrceugenia exsucca* trunk; **G.** *Ribes trilobum* growing on *Blepharocalyx cruckshanksii* main trunk crotch; **H.** *Lomatia ferruginea* growing on *Amomyrtus luma* main trunk crotch.

**Figure 4.**
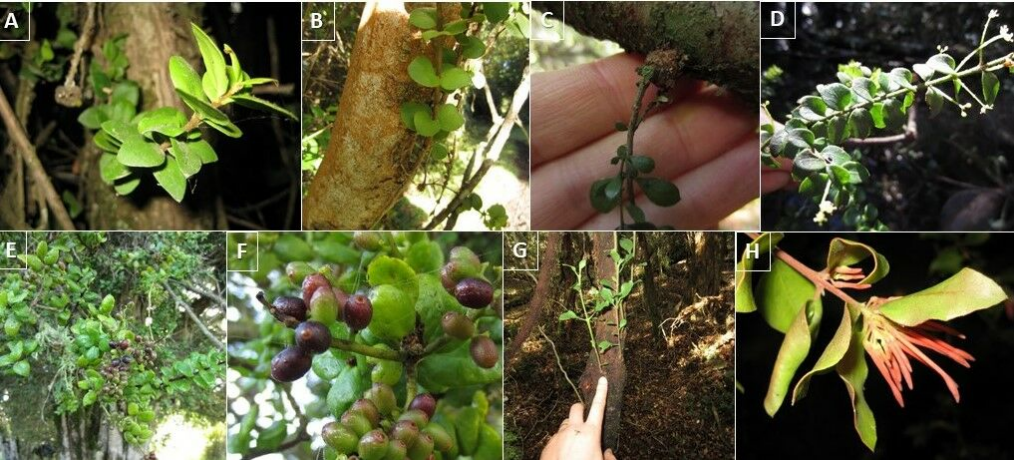
Hemiparasites: **A.** *Antidaphne punctulata* growing on *Myrceugenia exsucca*; **B.** *Antidaphne punctulata* growing on *Luma apiculata*; **C.** *Lepidoceras chilense*, insertion point on an *Blepharocalyx cruckshanksii* branch; **D.** *Lepidoceras chilense*, leaf distribution; **E.** *Notanthera heterophylla* growing on *Myrceugenia exsucca*; **F.** *Notanthera heterophylla*, details leaves and fruits; **G.** *Tristerix corymbosus* growing on climber *Cissus striata*; **H.** *Tristerix corymbosus*, details leaves and flower.

**Figure 5.**
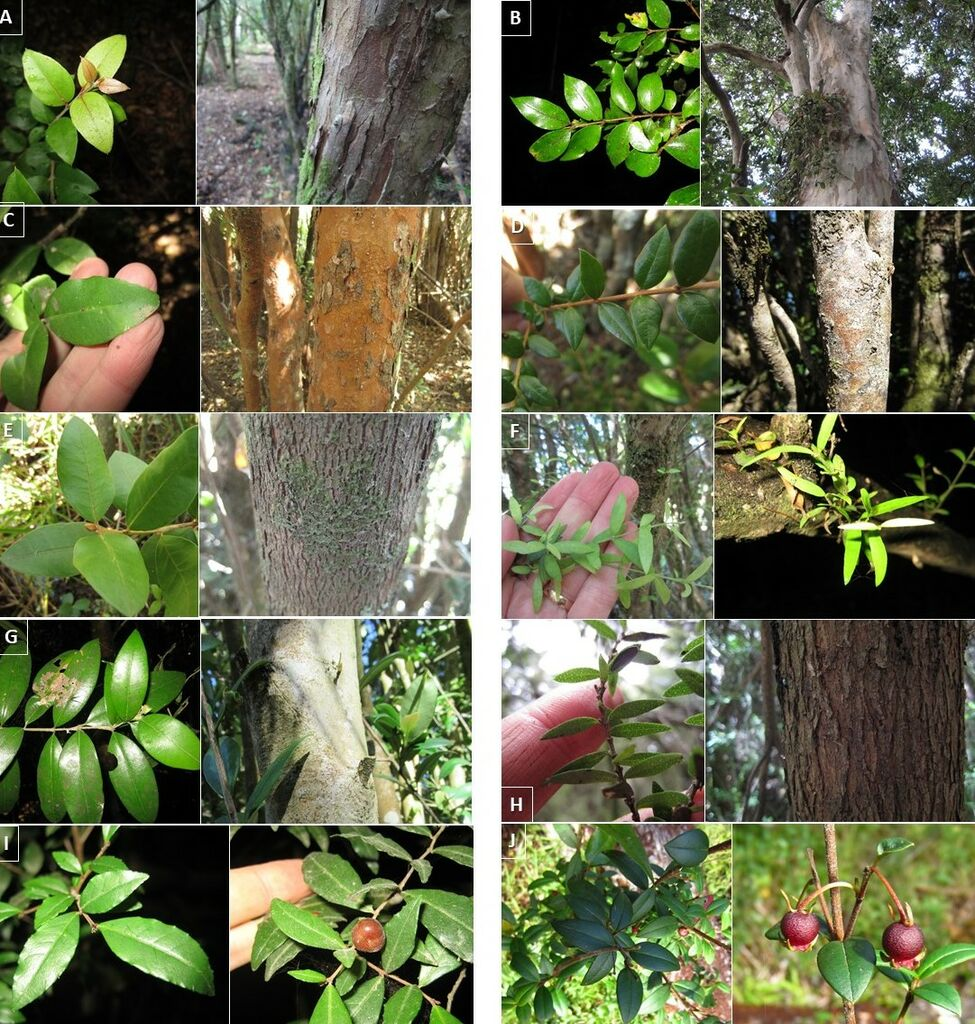
Common tree/shrub species. Leaves and bark: **A.** *Amomyrtus luma*; **B.** *Amomyrtus meli*; **C.** *Blepharocalyx cruckshanksii*; **D.** *Luma chequen*; **E.** *Myrceugenia exsucca*; **F.** *Myrceugenia parvifolia*; **G.** *Myrceugenia planipes*; **H.** *Tepualia stipularis*. Leaves and fruits (berries): **I.** *Rhamnus diffusus*; **J.** *Ugni molinae*.

In this contribution, we present a database of vascular plants in 30 swamp forest fragments distributed along the coast of the Araucanía Region (Table 1, Suppl. material 1). The data describes (i) species composition (Fig. 1) , (ii) condition (coarse woody debris, fallen log, live, snag), (iii) habit (herb, shrub, tree, Fig. 1), (iv) growth microhabitat (e.g. tree trunk, branch, main trunk crotch, fallen log, soil, Fig. 2), (v) growth form [accidental epiphyte (Fig. 3), hemiparasite (Fig. 4), terricolous (Fig. 5), vegetative], (vi) host species (as appropriate, Fig. 2) and (vii) relative location of the species in the sampled patch and surrounding areas (core, border, matrix). In addition, two introduced species were observed (*Cupressus macrocarpa* and *Prunus avium*) that are new to the catalogue of vascular plants of Chile (Rodríguez et al. 2018). Several of the biological backgrounds presented here have not been reported in literature (e.g. Hauenstein et al. 2002, [Bibr B6507055][Bibr B6507496]), so the database is left available in this manuscript. This contribution complements the work of Pincheira-Ulbrich et al. (2016) who reported the complete catalogue of climbing plants and vascular epiphytes in coastal Myrtaceae swamp forest in La Araucanía Region.

## General description

### Purpose

This contribution provides background information for biodiversity, meta-community or macro-ecological studies, as it also includes the geographical location of forest fragments. Some biodiversity traits have not been reported in literature, such as the recording of tree remains and accidental epiphytes. These data are expected to contribute to the local valuation and conservation of these highly-degraded ecosystems.

## Project description

### Study area description

The study area is located on the coast of the Araucanía Region of Chile (38°30′–39°30′S, 72°45′–73°30′W). It covers an area of 1656 km^2^, bounded by the Imperial River in the south and the Queule in the north and lying between the Coastal Range to the east and the Pacific Ocean to the west. The climate is oceanic with a Mediterranean influence, with average annual precipitation of 1200–1600 mm (Luebert and Pliscoff 2006).

The territory is distributed amongst numerous indigenous Mapuche communities and private farming/forestry properties (Pincheira-Ulbrich 2018). The land is divided into small plots, with high poverty and rural dwelling, mostly unchanged since the middle of the 20th century (Gissi 2004, Peña-Cortés et al. 2020). As a result, the historical and current pressure on the forest has meant that most of its area is in a degraded state, set in a matrix of anthropogenic landscape (Peña-Cortés et al. 2011, Hauenstein et al. 2014, Peña-Cortés et al. 2020b). Therefore, the forest is a secondary ecosystem (diameter at breast height of trees x = 19 ± 11 cm), consisting mainly of native species of the Myrtaceae family (10 species).

### Design description

The forest patches were grouped into five size classes: < 0.5 ha, 0.5–2 ha, 2–10 ha, 10–50 ha and > 50 ha. The seven largest fragments (> 50 ha) were chosen subjectively and six fragments were selected at random from each of the other classes, except the 0.5–2 ha class, which contained only five fragments. This produced a total of 30 sampling sites distributed over the whole study area (see Pincheira-Ulbrich et al. 2016). In the field, sampling design was non-random in order to include the largest possible variety of micro-habitats and rare species (Croft and Chow-Fraser 2009, Dieckman et al. 2007). Data were collected from 2011 to 2013, with 32 days spent in the field.

## Sampling methods

### Sampling description

Sampling followed a transect sampling observations protocol, orientated from the edge towards the centre of the fragment (Brower et al. 1990). Field notes and photographs, taken throughout the transect, were reviewed in the lab. Seven types of data were recorded: (i) Taxonomic identity, following the criteria established in the publications of Marticorena and Rodríguez (Marticorena and Rodríguez 2001, Marticorena and Rodríguez 2003, Marticorena and Rodríguez 2005, Marticorena and Rodríguez 2011), (ii) condition (coarse woody debris, fallen log, live, snag), according to Enrong et al. (2006), (iii) habit (herb, shrub, tree) according to Harris and Harris (2001), (iv) growth microhabitat (e.g. tree trunk, branch, main trunk crotch, fallen log soil) according to field observations, (v) growth form (accidental epiphyte, hemiparasite, terricolous, vegetative) according to Benzing (2008), (vi) host species (as appropriate) and (vii) relative location of the species in the sampled patch and surrounding areas (core, border, matrix). The taxonomic nomenclature was based on Rodríguez et al. (2018) and The International Plant Names Index (2019).

## Geographic coverage

### Description

The study area is located on the coast of the Araucanía Region of Chile (38°30′–39°30′S, 72°45′–73°30′W). It covers an area of 1656 km^2^, bounded by the Imperial River in the south and the Queule in the north and lying between the Coastal Range to the east and the Pacific Ocean to the west.

### Coordinates

Imperial River and Tolten Rive Latitude; Pacific Ocean and Coastal mountain range. Longitude.

## Traits coverage

Trees, shrubs, accidental epiphytes, host.

## Temporal coverage

### Notes

2011-2013

## Usage licence

### Usage licence

Creative Commons Public Domain Waiver (CC-Zero)

## Data resources

### Data package title

Trees, shrubs and herbs of the coastal Myrtaceae swamp forest in La Araucanía: a dataset

### Number of data sets

1

### Data set 1.

#### Data set name

Trees, shrubs and herbs of the coastal Myrtaceae swamp forest in La Araucanía: a dataset

#### Data format

csv

#### Number of columns

20

#### Data format version

csv

#### Description

The dataset provides a record of 55 species (24 trees, 1 vine, 16 herbs, and 15 shrubs) including accidental epiphytes (n = 6), hemiparasites (n = 4), host (n = 11) and additionally woody debris (n = 36) in 356 records. The data describes (i) species composition, (ii) condition (coarse woody debris, fallen log, live, snag), (iii) habit (herb, shrub, tree), (iv) growth microhabitat (e.g. tree trunk, branch, main trunk crotch, fallen log, soil), (v) growth form (accidental epiphyte, hemiparasite, terricolous, vegetative), (vi) host species (as appropriate) and (vii) relative location of the species in the sampled patch and surrounding areas (core, border, matrix). Several of the biological backgrounds presented here have not been reported in literature, so the database is left available in this manuscript.

**Data set 1.** 

Column label	Column description
Patch size (ha)	Forest fragment size in hectares.
Latitude	Geographic coordinate that specifies the north–south position of a point on the Earth's surface
Longuitude	Geographic coordinate that specifies the east–west position of a point on the Earth's surface
ID	Record number
Species	Scientific name of species
Condition	Living trees and tree debris. Coarse woody debris, Fallen log, Live, Snag
Habit	Growth habit according to literature. Herb, Shrub, Tree, NA (Not applicable)
Microhabitat	Site where the individual was observed growing. Base of trunk, Branch, Fallen log, Main trunk crotch, Soil, Stem, Trunk
Growth form	Growth form observed in the field. Accidental ephyphyte, Hemiparasite, Terricolous, Vegetative, NA (Not applicable)
Host	Scientific name of species.
Location1	Relative location 1 of the record in the field. Core, Core-Gap, Edge, Gap-Edge, Matrix.
CoordinateUncertaintyInMetres1	Horizontal distance (in metres) from the given decimal Latitude and decimal Longitude describing the smallest circle containing the whole of the Location.
Location2	Relative location 2 of the record in the field. Core, Core-Gap, Edge, Gap-Edge, Matrix, NA (Not applicable)
CoordinateUncertaintyInMetres2	Horizontal distance (in metres) from the given decimal Latitude and decimal Longitude describing the smallest circle containing the whole of the Location.
Location3	Relative location 3 of the record in the field. Core, Core-Gap, Edge, Gap-Edge, Matrix, NA (Not applicable)
CoordinateUncertaintyInMetres3	Horizontal distance (in metres) from the given decimal Latitude and decimal Longitude describing the smallest circle containing the whole of the Location.
Date	Registration date.
Sampling protocol	Field sampling protocol.
Observer name	Name of person who collected data in the field.
Notes	Other observations in the field, UD (Undefined)

## Supplementary Material

40B11CD0-9AE9-586D-95F6-345CDB9264B3 10.3897/BDJ.9.e63634.suppl1 Supplementary material 1 Trees, shrubs and herbs of the coastal Myrtaceae swamp forest in La Araucanía: a dataset Data type Occurrences Brief description The dataset provides a record of 55 species (24 trees, 1 vine, 16 herbs and 15 shrubs) including accidental epiphytes (n = 6), hemiparasites (n = 4), host (n = 11) and additionally woody debris (n = 36) in 357 records. The data describe (i) species composition, (ii) condition (coarse woody debris, fallen log, live, snag), (iii) habit (herb, shrub, tree), (iv) growth microhabitat (e.g. tree trunk, branch, main trunk crotch), (v) growth form (accidental epiphyte, hemiparasite, terricolous, vegetative), (vi) host species (as appropriate) and (vii) relative location of the species in the sampled patch and surrounding areas (core, border, matrix). Several of the biological backgrounds presented here have not been reported in literature, so the database is left available in this manuscript. File: oo_511976.csv https://binary.pensoft.net/file/511976 Jimmy Pincheira-Ulbrich, Elías Andrade Mansilla, Fernando Peña-Cortés, Cristian Vergara Fernández

## Figures and Tables

**Table 1.** Trees, shrubs and herbs of the Myrtaceae swamp forest. Classification of species according to the criteria of Phyllum, Family, Habitat and Geographical origin is based on Rodríguez et al. (2018).

**n**	**Specie**	**Phylum**	**Family**	**Habit**	**Geographic origin**
1	*Acacia melanoxylon* R. Br.	Magnoliophyta	Fabaceae	Tree	Introduced
2	*Acaena* spp.	Magnoliophyta	Rosaceae	Herb	Native
3	*Aextoxicon punctatum* Ruiz & Pav.	Magnoliophyta	Aextoxicaceae	Tree	Native
4	*Amomyrtus luma* (Molina) D. Legrand & Kause	Magnoliophyta	Myrtaceae	Tree	Native
5	*Amomyrtus meli* (Phil.) D. Legrand & Kausel	Magnoliophyta	Myrtaceae	Tree	Endemic
6	*Antidaphne punctulata* (Clos) Kuijt	Magnoliophyta	Santalaceae	Shrub	Endemic
7	*Aristotelia chilensis* (Molina) Stuntz	Magnoliophyta	Elaeocarpaceae	Tree	Native
8	*Berberis actinacantha* Mart.	Magnoliophyta	Berberidaceae	Shrub	Endemic
9	*Blechnum chilense* (Kaulf.) Mett.	Pterophyta	Blechnaceae	Herb	Native
10	*Blechnum hastatum* Kaulf.	Pterophyta	Blechnaceae	Herb	Native
11	*Blepharocalyx cruckshanksii* (Hook. & Arn.) Nied.	Magnoliophyta	Myrtaceae	Tree	Endemic
12	*Caldcluvia paniculata* (Cav.) D. Don	Magnoliophyta	Cunoniaceae	Tree	Native
13	*Chusquea quila* Kunth	Liliopsida	Poaceae	Herb	Endemic
14	*Cissus striata* Ruiz & Pav.	Magnoliophyta	Vitaceae	Vine	Native
15	*Cupressus macrocarpa* Hartw	Pinophyta	Cupressaceae	Tree	Introduced
16	*Dioscorea auriculata* Poepp.	Liliopsida	Dioscoreaceae	Herb	Endemic
17	*Drimys winteri* J.R. Forst. & G. Forst.	Magnoliophyta	Winteraceae	Tree	Endemic
18	*Eucryphia cordifolia* Cav.	Magnoliophyta	Eucryphiaceae	Tree	Native
19	*Fuchsia magellanica* Lam.	Magnoliophyta	Onagraceae	Shrub	Native
20	*Galium hypocarpium* (L.) Endl. ex Griseb.	Magnoliophyta	Rubiaceae	Herb	Native
21	*Gaultheria phillyreifolia* (Pers.) Sleumer	Magnoliophyta	Ericaceae	Shrub	Native
22	*Gavilea* spp.	Liliopsida	Orchidaceae	Herb	Native
23	*Greigia sphacelata* (Ruiz & Pav.) Regel	Liliopsida	Bromeliaceae	Herb	Native
24	*Hieracium* spp.	Magnoliophyta	Asteraceae	Herb	UD
25	*Hydrocotyle poeppigii* DC.	Magnoliophyta	Apiaceae	Herb	Endemic
26	*Juncus* spp.	Liliopsida	Juncaceae	Herb	Native
27	*Lepidoceras chilense* (Molina) Kuijt	Magnoliophyta	Santalaceae	Shrub	Endemic
28	*Lomatia ferruginea* (Cav.) R. Br.	Magnoliophyta	Proteaceae	Tree	Native
29	*Lophosoria quadripinnata* (J.F. Gmel.) C. Chr.	Pterophyta	Dicksoniaceae	Herb	Native
30	*Luma apiculata* (DC.) Burret	Magnoliophyta	Myrtaceae	Tree	Native
31	*Luma chequen* (Molina) A. Gray	Magnoliophyta	Myrtaceae	Tree	Endemic
32	*Maytenus boaria* Molina	Magnoliophyta	Celastraceae	Tree	Native
33	*Myrceugenia exsucca* (DC.) O. Berg	Magnoliophyta	Myrtaceae	Tree	Native
34	*Myrceugenia parvifolia* (DC.) Kausel	Magnoliophyta	Myrtaceae	Shrub	Endemic
35	*Myrceugenia planipes* (Hook. & Arn.) O. Berg	Magnoliophyta	Myrtaceae	Tree	Native
36	*Myriophyllum aquaticum* (Vell.) Verdc.	Magnoliophyta	Haloragaceae	Herb	Native
37	*Nertera granadensis* (Mutis ex L.f.) Druce	Magnoliophyta	Rubiaceae	Herb	Native
38	*Notanthera heterophylla* (Ruiz & Pav.) G. Don	Magnoliophyta	Loranthaceae	Shrub	Endemic
39	*Nothofagus dombeyi* (Mirb.) Oerst.	Magnoliophyta	Nothofagaceae	Tree	Native
40	*Nothofagus obliqua* (Mirb.) Oerst.	Magnoliophyta	Nothofagaceae	Tree	Native
41	*Ovidia pillo-pillo* (Gay) Meisn.	Magnoliophyta	Thymelaeaceae	Shrub	Endemic
42	*Persea lingue* (Ruiz & Pav.) Nees	Magnoliophyta	Lauraceae	Tree	Native
43	*Prunus avium* (L.) L.	Magnoliophyta	Rosaceae	Tree	Introduced
44	*Rhamnus diffusus* Clos	Magnoliophyta	Rhamnaceae	Shrub	Endemic
45	*Rhaphithamnus spinosus* (Juss.) Moldenke	Magnoliophyta	Verbenaceae	Shrub	Native
46	*Ribes trilobum* Meyen	Magnoliophyta	Grossulariaceae	Shrub	Endemic
47	*Rubus constrictus* P.J. Müll. & Lefèvre	Magnoliophyta	Rosaceae	Shrub	Introduced
48	*Salix babylonica* L.	Magnoliophyta	Salicaceae	Tree	Introduced
49	*Salix caprea* L.	Magnoliophyta	Salicaceae	Tree	Introduced
50	*Sophora cassioides* (Phil.) Sparre	Magnoliophyta	Fabaceae	Tree	Endemic
51	Sp1	Liliopsida	Cyperaceae	Herb	Native
52	*Tepualia stipularis* (Hook. & Arn.) Griseb	Magnoliophyta	Myrtaceae	Tree	Native
53	*Tristerix corymbosus* (L.) Kuijt	Magnoliophyta	Loranthaceae	Shrub	Native
54	*Ugni molinae* Turcz.	Magnoliophyta	Myrtaceae	Shrub	Native
55	*Ulex europaeus* L.	Magnoliophyta	Fabaceae	Shrub	Introduced
